# Longitudinal Spin Seebeck Effect Thermopiles Based on Flexible Co-Rich Amorphous Ribbons/Pt Thin-Film Heterostructures

**DOI:** 10.3390/s23187781

**Published:** 2023-09-10

**Authors:** Marcio A. Correa, Andrey V. Svalov, Armando Ferreira, Matheus Gamino, Edimilson F. da Silva, Felipe Bohn, Filipe Vaz, Danniel F. de Oliveira, Galina V. Kurlyandskaya

**Affiliations:** 1 Departamento de Física, Universidade Federal do Rio Grande do Norte, Natal 59078-900, RN, Brazil; marciocorrea@fisica.ufrn.br (M.A.C.); mgamino@fisica.ufrn.br (M.G.); efelix@fisica.ufrn.br (E.F.d.S.); felipebohn@fisica.ufrn.br (F.B.); 2 Centro de Física das Universidades do Minho e do Porto (CF-UM-UP), Universidade do Minho, 4710-057 Braga, Portugal; armando.f@fisica.uminho.pt (A.F.);; 3 Institute of Natural Sciences and Mathematics, Ural Federal University, 620002 Ekaterinburg, Russia; andrey.svalov@urfu.ru; 4 LaPMET—Laboratório de Física para Materiais e Tecnologias Emergentes, Universidade do Minho, 4710-057 Braga, Portugal; 5 Departamento de Ciências dos Materiais, Universidade Federal da Paraíba, João Pessoa 58059-900, PB, Brazil; danniel.oliveira@acadmico.ufpb.br

**Keywords:** anomalous Nernst effect, spintronics, thermoelectric conversion, flexible magnetic materials, magnetic properties, magnetic sensors

## Abstract

Thermoelectric phenomena, such as the Anomalous Nernst and Longitudinal Spin Seebeck Effects, are promising for sensor applications in the area of renewable energy. In the case of flexible electronic materials, the request is even larger because they can be integrated into devices having complex shape surfaces. Here, we reveal that Pt promotes an enhancement of the thermoelectric response in Co-rich ribbon/Pt heterostructures due to the spin-to-charge conversion. Moreover, we demonstrated that the employment of the thermopiles configuration in this system increases the induced thermoelectric current, a fact related to the considerable decrease in the electric resistance of the system. By comparing present findings with the literature, we were able to design a flexible thermopile based on LSSE without the lithography process. Additionally, the thermoelectric voltage found in the studied flexible heterostructures is comparable to the ones verified for rigid systems.

## 1. Introduction

Co-rich amorphous ribbons have excellent ferromagnetic properties. They can be employed in a broad range of technological applications, including wound transformers, inductors, magnetic shielding [1,2], and high-frequency magnetic sensing devices [3,4,5,6]. In recent years, they were also extensively studied with focus on biomedical applications for sensitive elements of magnetoimpedance-based devices for both label-free and magnetic label detection [7]. The amorphous Co-rich alloys often present high saturation magnetization, high magnetic permeability, and low magnetic hysteresis losses [8,9,10]. Furthermore, the flexibility of these materials in combination with the corrosion stability of Cr-doped compositions makes them promising candidates for sensor applications, particularly for covering of the curved surfaces or surfaces having even more complex geometry. All these features are interesting for thermoelectric applications based on the Longitudinal Spin Seebeck Effect (LSSE) [11,12,13] and Anomalous Nernst Effect (ANE) [14,15,16,17].

While there is a limited number of experimental works examining thermoelectric effects in ferromagnetic ribbons [18,19], the existing literature presents promising findings. Recently, we have explored the ANE effect in Fe_3_Co_67_Cr_3_Si_15_B_12_ amorphous ribbons [18], disclosing the influence of the relaxation and annealing under stress of the amorphous samples on their thermoelectric response. In particular, for temperature differences of ΔT∼22 K, thermoelectric signals of about 30 μV were achieved. However, it is worth mentioning that to reach such ΔT levels, the top of the ribbon may often be heated up to the high temperatures of about 80 °C. Considering that the Co-rich ribbons are not protected with a cap layer, surface oxidation can take place. Therefore, this issue must be considered for future sensor applications, especially if long-time stability is expected [20].

It is well known that an in-vacuum annealing process can be used for the improvement of the magnetic properties of the ribbons. This is not the case of the heating in thermoelectric experiments, such as LSSE and ANE measurements, that are carried out in air, hence inducing high levels of oxidation in the ribbons. A possible solution to overcome this issue consists of the use of the protecting covering layer. While the deposition of an insulating layer appears, in some aspects, as an interesting alternative for protection, it drastically modifies the electrical properties of the surface, making the thermoelectric measurements a hard task. In this sense, the usage of metallic protecting layers with high corrosion stability is preferred. Additionally, some specific metallic layers allow the amplification of the thermoelectric signal when a heterostructure ribbon/metallic layer is considered. This is the case for materials with high spin-orbit coupling and positive spin hall angle, which promote a raise in the spin-to-charge current conversion. Remarkably, Platinum (Pt) seems to be the best solution for both protecting against oxidation [21,22,23,24,25,26] and promoting spin-to-charge conversion [27,28,29].

From the theoretical point of view, both ANE and LSSE have similar descriptions, in which a magnetic material is submitted to a thermal gradient $\vec{\nabla }T$. As a consequence, an electric field $\vec{E}$ is induced and can be detected as a thermoelectric voltage V. Despite this similarity, the origin of these two effects is fundamentally different. The ANE electrical field comes from the relation between the magnetization of the material and the thermal gradient, and it is given by [29,30],

(1) $${\vec{E}}_{ANE}=-S_{ANE}\left(\vec{m}\times \vec{\nabla }T\right),$$

where $\vec{m}$ is the magnetization vector with saturation magnetization $m_{s}$, and $S_{ANE}$ is the Anomalous Nernst Effect coefficient, which brings to light the effective efficiency of the thermoelectric conversion in the magnetic system. The LSSE is in turn connected to the generation of pure spin current, which is converted into charge current through the Inverse Spin Hall Effect (ISHE),

(2) $${\vec{J}}_{c}=\frac{2e}{\hbar }\theta_{SH}\left({\vec{J}}_{s}\times \hat{\sigma }\right),$$

in which ${\vec{J}}_{c}$ is the charge current, e is the elementary charge, ℏ corresponds to the Planck constant divided by 2π, $\theta_{SH}$ is the spin Hall angle, $\hat{\sigma }$ represents the magnetic polarization, and finally ${\vec{J}}_{S}$ is the spin current that flows to a metallic layer placed onto the magnetic one. Phenomenologically, the LSSE electrical field can be written as

(3) $${\vec{E}}_{LSSE}=-S_{LSSE}\left(\hat{\sigma }\times \vec{\nabla }T\right),$$

where $S_{LSSE}$ is the LSSE coefficient.

Experimentally, $\hat{\sigma }$ can be changed by the application of an external magnetic field $\vec{H}$, which modifies the magnetization $\vec{m}$ of the material. Taking a closer look at Equations (1) and (3), one can notice a strict relation between both effects. The consequence is that, for a *metallic* ferromagnetic/Pt heterostructure, both effects are accessed during the thermoelectric experiment, bringing the contributions of ANE and LSSE simultaneously. While some studies found in the literature have focused on the separation of such contributions [31], for the case of metallic ribbon/Pt heterostructures, we concentrate on obtaining reliable estimates of an effective thermoelectric coefficient $S_{eff}$.

At the same time, for a practical application, it is necessary to improve the thermoelectric signal of the system. In this sense, design thermopile seems to be a promising path for the increase of the electrical signal. This geometry is explored in distinct studies in the literature [32,33,34,35], in which the magnetic system is deposited onto rigid substrate and/or produced using lithography processes. For instance, recently, Weng et al. [35] demonstrated the efficiency on the ANE thermopile composed by a single ferromagnetic element. In this study they showed a linear increase of the thermoelectric signal as the length of the thermopile increases. Kim et al. [33] in turn presented a spin thermopile system in which exchange bias Pt/CoFeB multilayers are explored. In the results, the authors demonstrated a linear increase of thermoelectric signal with the number of bilayers. In the same sense, Uchida et al. [32] used Pt/Nb thermopile based on Y_3_Fe_5_O_13_ (YIG) to induce an improvement of the thermoelectric signal of the proposed system. From the results, a remarkable increase in the induced thermoelectric voltage of Pt/Nb thermopile in comparison with a single YIG/Pt system was observed.

In the present study, we designed, developed, and comparatively analyzed bare Co-rich ribbon and ribbon/Pt heterostructures and explored the Anomalous Nernst Effect and Longitudinal Spin Seebeck Effect showing that the Pt cap layer promotes an enhancement of the thermoelectric response. Further, we demonstrated the advantages of designing thermopiles consisting of Co-rich ribbon/Pt heterostructures in a parallel association, which can considerably improve the thermoelectric current response.

## 2. Materials and Methods

**Figure 1:**
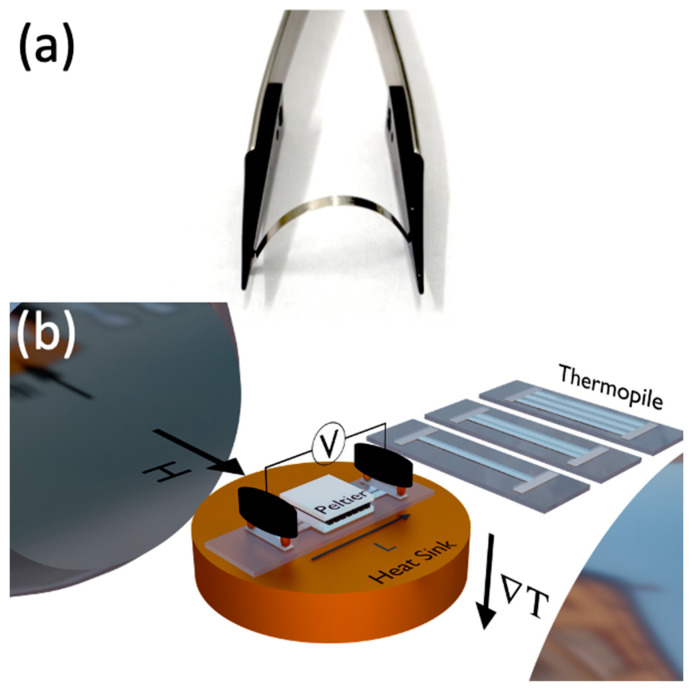
(**a**) Image of bare Fe_3_Co_67_Cr_3_Si_15_B_12_ flexible ribbon. (**b**) Schematic representation of the thermoelectric experimental setup. In particular, the arrow characterizing the field corresponds to the direction labeled as $\varphi_{H}=90{}^{\circ}$. We show the sketch of the thermopiles explored in this study (Pt, Pt2, Pt3 for one, two, and three ribbons, respectively).

We investigated flexible Fe_3_Co_67_Cr_3_Si_15_B_12_ (self-fabricated by the authors at UPV-EHU, Leioa, Spain) amorphous ribbons with a thickness of $t_{F}\approx 0.24$ μm and width of w≈0.8 mm (Figure 1a). According to the existing studies, the ribbons of this composition have a very small negative magnetostriction coefficient λ_s_ = −1 × 10^−7^ [36,37]. For this reason, we expect that the thermoelectric response does not show considerable change with stress applications.

The samples were prepared by a rapid quenching technique, following a procedure similar to that described in Refs. [18,37,38], thus obtaining ribbons with a length of up to 10 m. Amorphous ribbons were prepared by the melt-spinning technique onto a rotating roller. The master alloy of desired composition was melted in a quartz crucible using an inductor coil and ejected by Ar pressure jump onto a copper roller rotating at 470 rad/s in a vacuum chamber. High-quality ribbons with shine-free surfaces were obtained.

Here, no annealing was performed to modify the structure and magnetic properties of the amorphous as-cast ribbons. To cap the ribbons, the Magnetron Sputtering technique was employed. Specifically, we considered a 6 nm thick Platinum layer as a cap layer, deposited with the following experimental parameters: base pressure of $7\times 10^{-8}$ Torr, deposition pressure of $3\times 10^{-3}$ Torr with 20 sccm Ar flow, and 50W set in the DC source, resulting in a deposition rate of 0.98 Å/s. During the deposition, the ribbons were kept in a constant rotation to avoid any anisotropy induction due to the residual magnetic field of the magnetron sputtering guns. In particular, the Pt thickness was carefully chosen, allowing the achievement of good efficiency in the spin-charge current during the LSSE measurements [39].

Regarding the characterization of the samples, first magnetization curves at room temperature were obtained using a Vibrating Sample Magnetometer (VSM, Lake Shore 7404, Westerville, OH, USA). Amorphous ribbons of 13 mm lengths were employed. Experiments were performed with the magnetic field applied along the main axis to the ribbon ($\varphi_{H}=0{}^{\circ}$) and perpendicular to the long side in-plane of the ribbon ($\varphi_{H}=90{}^{\circ}$). The thermoelectric voltage measurements were obtained using a homemade system. In this case, the thermal gradient ∇T was applied by using a micro-Peltier module (see Figure 1b), which heats the top of the heterostructure. At the same time, the bottom of the glass substrate was put in thermal contact with a heat sink. In particular, a glass substrate was considered to avoid the electrical contact of the sample with the metallic heat sink. To improve thermal conductivity, thermal paste was used in the system. The voltage detection was done with silver conductive glue and using gold spring contacts distanced L=11 mm from each other. From theory [30], the thermoelectric voltage is described as follows:

(4) $$V=-\int_{0}^{L}\vec{E}\cdot d\vec{l},$$

where $d\vec{l}$ is the length differential element integrated along the distance between electrical contacts L. The magnetic field is applied through an electromagnet controlled by using a Kepco bipolar source (BOP 20/20). The thermoelectric signal is measured by using a high-precision $6\frac{1}{2}$ digits multimeter (Keithley). The sample rotation was performed by a high-resolution step motor. All the experiments were controlled through a homemade LabView software (LabVIEW Free online IDE for Learner). During the thermoelectric measurements, each experimental point was an average of 10 measurements. A similar procedure was employed for the magnetic characterization, in which each experimental point was an average of 20 measurements. For this reason, we believe that the experimental results reflect the real efficiency of the proposed thermopile in these specific experimental conditions (room temperature, controlled humidity and pressure).

The experiments were performed using both the bare Fe_3_Co_67_Cr_3_Si_15_B_12_ amorphous ribbon and the Fe_3_Co_67_Cr_3_Si_15_B_12_/Pt heterostructure for comparative ANE and ANE + LSSE responses. Prototypes with one (Pt), two (Pt2), and three (Pt3) Fe_3_Co_67_Cr_3_Si_15_B_12_/Pt heterostructures in a parallel configuration were tested. The Fe_3_Co_67_Cr_3_Si_15_B_12_/Pt heterostructures connected by using conductive silver glue, onto a glass substrate, are depicted in Figure 1b.

## 3. Results and Discussion

**Figure 2:**
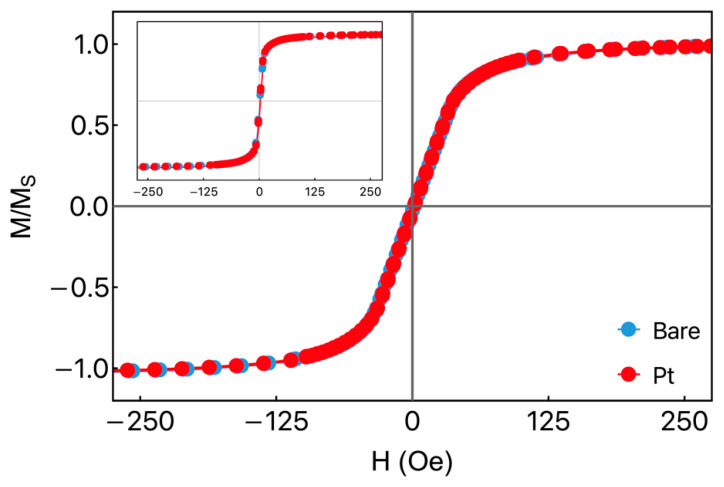
Normalized magnetization curve as a function of the external magnetic field taken at $\varphi_{H}=90{}^{\circ}$ for the bare ribbon and Fe_3_Co_67_Cr_3_Si_15_B_12_/Pt heterostructure. In the inset, we show the corresponding magnetization response for $\varphi_{H}=0{}^{\circ}$.

Figure 2 shows the magnetization curves for the bare ribbon (without an additional cap layer) and the Fe_3_Co_67_Cr_3_Si_15_B_12_/Pt heterostructure with the external magnetic field applied along the $\varphi_{H}=90{}^{\circ}$ and 0° directions. Remarkably, for each magnetic field orientation, the curves measured for the bare ribbon and heterostructure are similar. It is an indicator that the presence of the Pt layer in the heterostructure does not affect the magnetic response of the system. Nevertheless, we find differences in the magnetization curves when the responses for distinct field orientations are compared. For both samples, the coercive field $H_{c}$ was close to 1.2 Oe, while the saturation field $H_{s}$ was 35 Oe and 125 Oe when the experiments were performed at $\varphi_{H}=0{}^{\circ}$ and 90°, respectively. It is interesting to notice that the rotation of the field does not cause significant changes in the coercive field but leads to strong modifications in the saturation field of the samples. This observation suggests that the effective magnetic anisotropy is longitudinal and primarily influenced by the shape of the samples. These findings are in concordance with previous studies reported in the literature [18].

**Figure 3:**
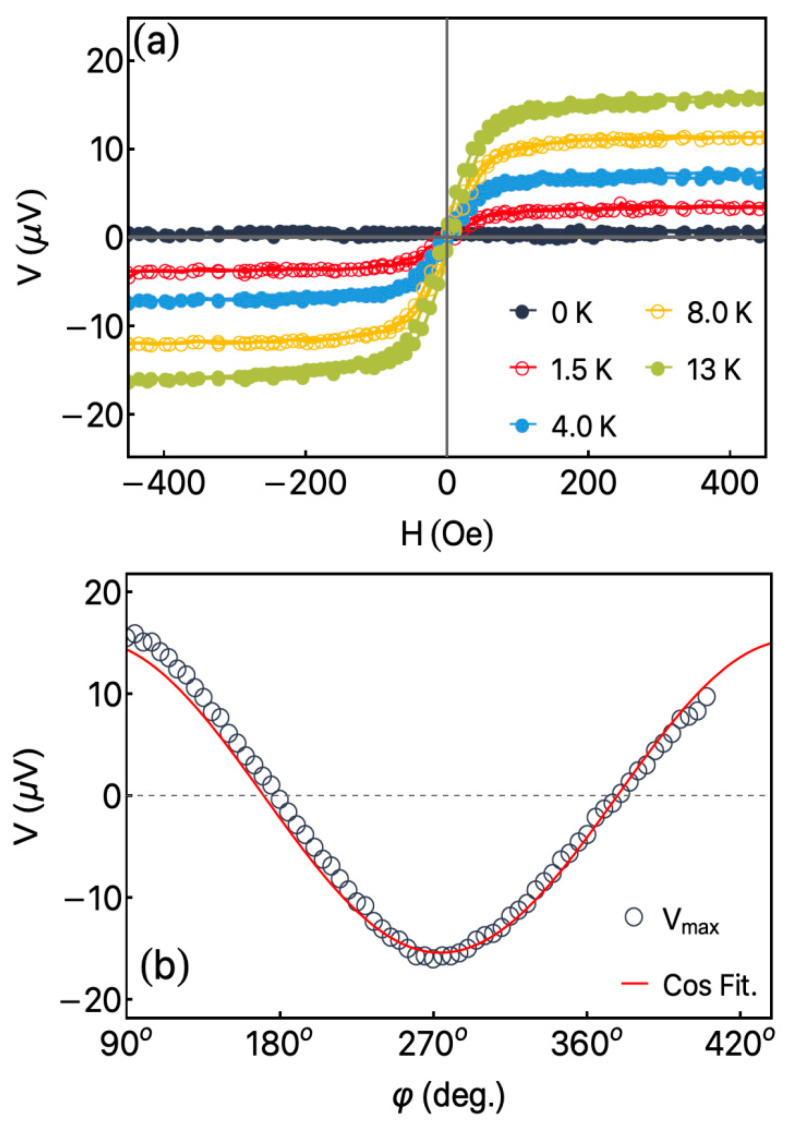
(**a**) Thermoelectric current as a function of the external magnetic field for selected values of the temperature difference ΔT for the bare Fe_3_Co_67_Cr_3_Si_15_B_12_ ribbon. The measurements were obtained for $\varphi_{H}=90{}^{\circ}$. This configuration maximizes the induced electric field and, consequently, the thermoelectric voltage/current, as depicted in Equation (1). (**b**) Angular dependence of the $I_{max}$ for the bare ribbon. The solid line is the expected behavior predicted by Equations (1), (2) and (4) for a magnetically saturated system.

Figure 3 shows the thermoelectric response for the bare Fe_3_Co_67_Cr_3_Si_15_B_12_ ribbon. The thermoelectrical measurements were carried out varying the external magnetic field H and temperature difference ΔT between the top of the sample and the bottom of the glass substrate. For the bare ribbon, the sample structure allows us to assess uniquely the ANE. From Figure 3a, we disclose the thermoelectric voltage as a function of the external magnetic field for selected values of the temperature difference ΔT. The field alignment was set to $\varphi_{H}=90{}^{\circ}$ to improve the thermoelectric signal. At $\varphi_{H}=90{}^{\circ}$, the electrical field $\vec{E}$ is induced in the same direction of the electrical contacts $\vec{L}$ (see Figure 1b). For ΔT = 0 K, we observe no thermoelectric signal, as expected. Nevertheless, thermoelectric voltage increases linearly as ΔT rises. Moreover, it is possible to observe the connection between the thermoelectric and magnetization curves, irrespective of the ΔT value. From Figure 3b, in turn, we show the thermoelectric voltage as a function of the magnetic field angle, at ΔT = 13 K, and external magnetic field intensity of H = 400 Oe, where the sample is saturated magnetically. This behavior is expected, since for fixed ΔT and H values, Equations (1), (3) and (4) lead to a sine/cosine-shaped curve [29,30]. These initial results suggested the use of thermopiles configuration to investigate the response of Fe_3_Co_67_Cr_3_Si_15_B_12_/Pt heterostructures and the role of the Pt cap layer in the thermoelectric effects, as well as exploring a thermopiles configuration.

Regarding the sample structure, the deposition of the Pt onto the ribbon allows us to assess both LSSE and ANE phenomena. Here, it is worth remarking that Platinum has a large and positive spin Hall angle $\theta_{SHE}$, so an increase in the thermoelectric response can be observed due to the spin-to-charge current conversion by ISHE. Moreover, the metallic Pt layer modifies the surface of the heterostructure, hence causing a decrease in the electric resistance and yielding an increase in the measured thermoelectric *current*. Therefore, in addition to protecting the ribbon surface against oxidation, the Pt layer can enhance the induced signal.

These initial results gave us the motivation to use thermopiles configuration to investigate the response of Fe_3_Co_67_Cr_3_Si_15_B_12_/Pt heterostructures and the role of the Pt cap layer on the thermoelectric effects, as well as explore a thermopiles configuration.

**Figure 4:**
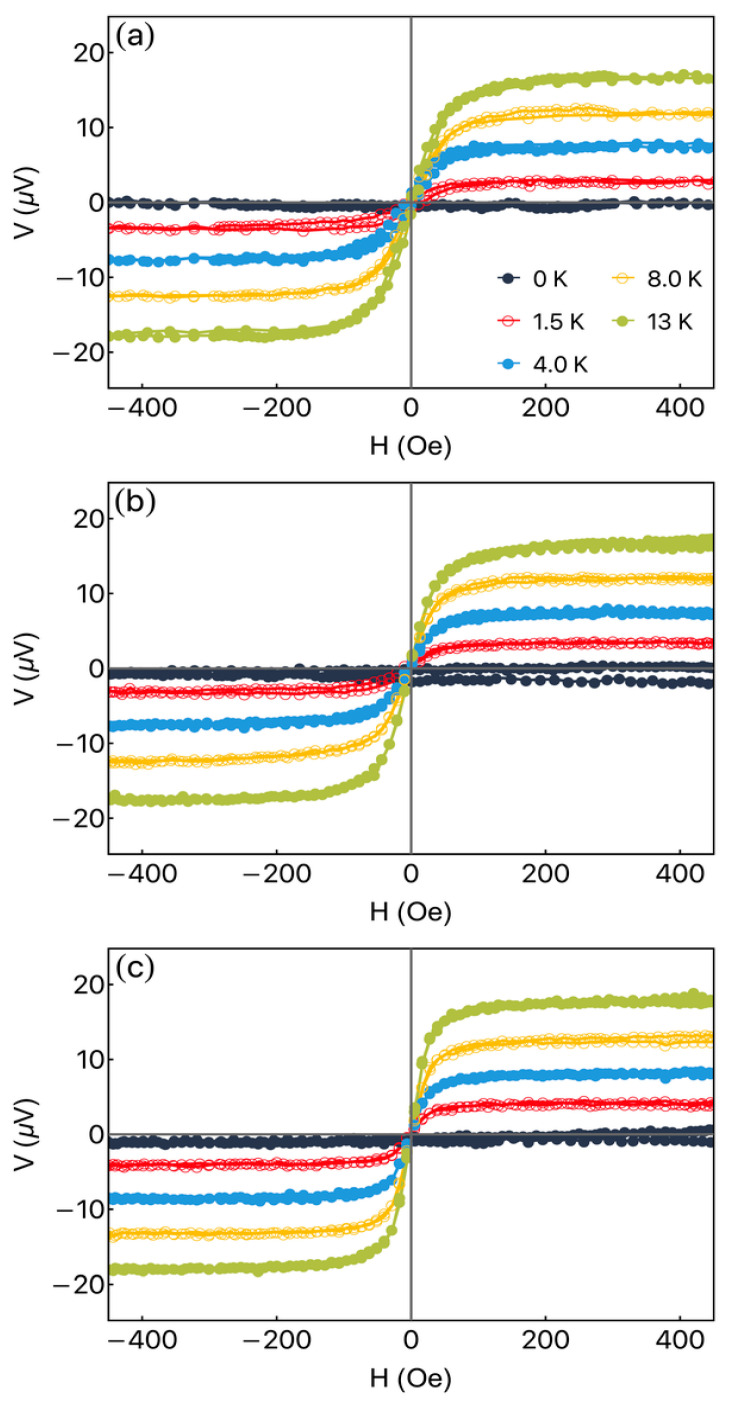
Thermoelectric response for the Fe_3_Co_67_Cr_3_Si_15_B_12_/Pt heterostructure. Specifically, we address the thermoelectric voltage as a function of the external magnetic field, at $\varphi_{H}=90{}^{\circ}$, for distinct ΔT values for the Fe_3_Co_67_Cr_3_Si_15_B_12_/Pt heterostructure, considering: (**a**) single thermopile (Pt); (**b**) double thermopile (Pt2); (**c**) triple thermopile (Pt3).

Figure 4 shows the thermoelectric response for the Fe_3_Co_67_Cr_3_Si_15_B_12_/Pt heterostructure. First of all, Figure 4a shows results for a single thermopile. The experimental setup with $\varphi_{H}=90{}^{\circ}$ induces again the mirroring of the magnetic and thermoelectric responses. From a general point of view, the thermoelectric behavior is similar to the one verified for the bare ribbon, as expected. Nevertheless, it is possible to verify a small increase in the thermoelectric current when the heterostructure is considered. This feature is a signature of the thermoelectric contribution from the Longitudinal Spin Seebeck Effect associated with spin-to-charge current conversion [30].

Looking at Figure 4a–c, one can see quite a similar evolution of the curves when different numbers of heterostructures composing the thermopiles are used. This behavior is expected once the parallel association of thermopiles does not modify the value of the induced voltage. On the other hand, from the point of view of the values of the thermoelectric current, the results are very promising, and the details of the discussion will be given below.

**Figure 5:**
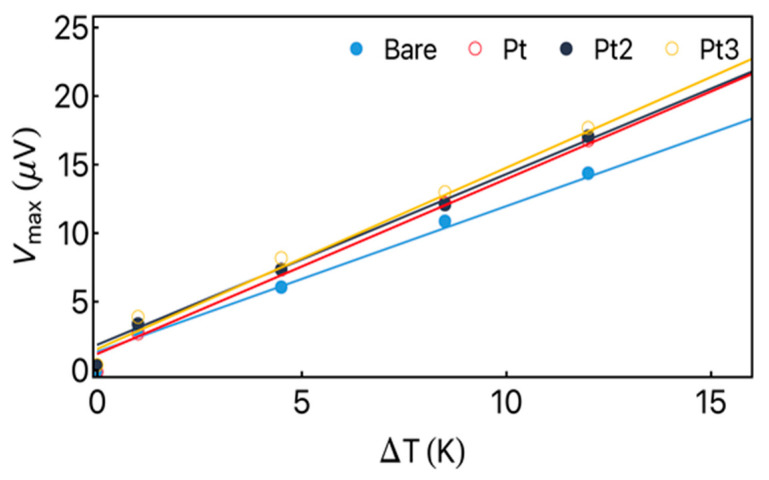
$V_{max}$ as a function of temperature difference ΔT measured during the experiment for the bare ribbon and for the heterostructures in the configurations of thermopiles. The solid line indicates the best linear fit of the data.

Figure 5 shows the maximum thermoelectric voltage $V_{max}$ as a function of the temperature difference ΔT achieved for the bare amorphous ribbon and Fe_3_Co_67_Cr_3_Si_15_B_12_/Pt heterostructure for a distinct number of thermopiles. The solid lines indicate the best linear fit for the experimental data. From the obtained results, it is possible to observe that the bare Fe_3_Co_67_Cr_3_Si_15_B_12_ ribbon has a slope smaller than the ones found for the Fe_3_Co_67_Cr_3_Si_15_B_12_/Pt heterostructures. Moreover, for all thermopiles, no significant modification of the slope is verified, which indicates that the effective thermoelectric coefficient $S_{eff}$ for these systems is similar, as expected. In particular, the slope of the obtained curves is the signature of the energy conversion efficiency for the designed heterostructure.

From the relation between $V_{max}$ and ΔT, we are able to calculate the effective thermoelectric coefficient $S_{eff}$ for each system. To this end, the temperature difference $\Delta T_{H}$ on the heterostructure was estimated. In the case under consideration, the material was magnetic. Experimentally, it is given by following equation [15,18]:

(5) $$\Delta T_{H}=\frac{t_{H}K_{s}}{t_{s}K_{H}}\Delta T,$$

where $t_{H}$ and $K_{H}$ are the thickness and thermal conductivity of the heterostructure, respectively, while $t_{s}$ and $K_{s}$ are corresponding quantities for the substrate. Then, from the dependence of $V_{max}$ with $\Delta T_{H}$, $S_{eff}$ is obtained from the equation:

(6) $$S_{eff}=\frac{t_{R}V_{max}}{L\Delta T_{H}}.$$

Then, assuming $t_{s}=0.15$ mm, $K_{s}=1.15$ W/mK [18], $K_{H}=133.79$ W/mK, and $t_{H}=0.24$ μm, we found the $S_{eff}$ values of around 1.75 μV/mK for the bare ribbon, and an average value of 2.01 μV/mK for the Fe_3_Co_67_Cr_3_Si_15_B_12_/Pt heterostructures. Notice the remarkable enhancement of $S_{eff}$ for such heterostructures, which is a signature of the overlap of the ANE and LSSE effects contributing to the thermoelectric voltage.

**Figure 6:**
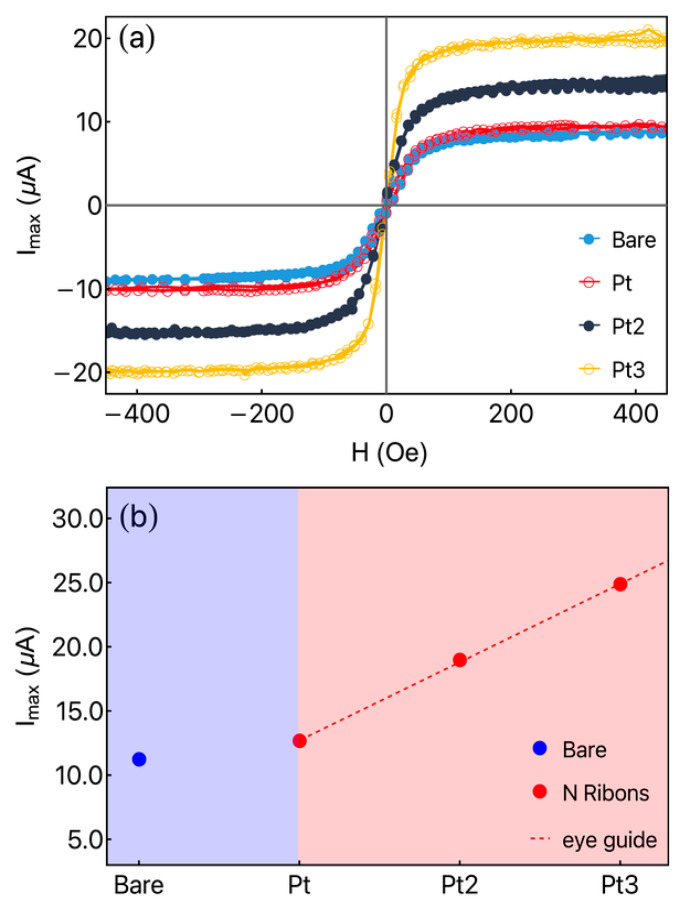
(**a**) The amplitude of the thermoelectric current as a function of the external magnetic field for the bare ribbon and thermopiles Pt, Pt2, and Pt3. (**b**) Maximum thermoelectric current for the bare ribbon (blue rectangle part) and thermopiles (red rectangle part). The dashed line is just a guide for the eyes.

Figure 6a brings to light the ratio of the thermoelectric *current* by the temperature difference as a function of the external magnetic field for the bare ribbon and thermopiles. For this purpose, we consider the remarkable decrease in the electrical resistance of the thermopile as the number of ribbon increases. In particular, here, we observed a decrease from R=1.76 Ω for Pt1 (single Fe_3_Co_67_Cr_3_Si_15_B_12_/Pt heterostructure) to R=1.18 Ω for Pt2, and R=0.90 Ω for Pt3.

Figure 6b shows the maximum $I_{max}$ for the bare ribbon (blue region) and thermopiles (red region). The significant increase of this parameter shows a promising path for exploring these Fe_3_Co_67_Cr_3_Si_15_B_12_/Pt heterostructures that are highly efficient for green energy devices. In the figure, the dashed line is an eye guide drawn using a linear function, as predicted by the increase of the current in an ohmic system. The observed behavior is mainly connected to the decrease in the electrical resistance in the thermopile when the parallel association is considered. It is important to point out that, if considered a parallel association of bare ribbons, a similar effect is reached.

Our findings bring to light the multifunctionality of the Pt cap layer in the system. Here, the Pt layer improves the protection of the ribbon against oxidation, leads to an increase in the thermoelectric voltage due to the spin-to-charge conversion, and decreases the electrical resistance of the heterostructure (1.90 Ω for the bare ribbon to 1.76 Ω for the heterostructure).

Obtained results are promising when compared with previous studies. The linear increase in the thermoelectric current associated with the number of ribbons in the thermopile is similar to the one found in the literature [32,33,34,35]. Moreover, we are able to reach values of around 20 μV for thermoelectric voltage, a value similar to those found in Ref. [35]. However, in our case, although the number of ribbons can limit the thermopile dimensions for a given application, no lithography process was required for the production process.

However, as aforementioned, flexible electronic materials become more requested in functional electronic units because they can be integrated into devices having complex shape surfaces. Flexibility of a magnetic field’s sensitive elements is a very attractive property for different types of magnetic sensors [38,40]. Although we did not take advantage of flexible design for thermopiles based on Co-rich amorphous ribbons/Pt thin-film heterostructures in the present study, it corresponds to an issue to be highlighted. The thermopiles exploring flexible ferromagnetic ribbons were studied in the present work. It is hard to make a quantitative comparison with other types of devices having thermoelectric responses. However, amorphous ribbons have much higher thermal conductivity in comparison with any flexible polymer substrate, and, therefore, the devices based on them may have an advantage of symmetry of thermoelectric response. Certainly, it is a promising direction for research and applications in the future. In addition, a large variety of functional cover layers can be considered in a search for the particular performances requested by the applications. The present findings can motivate new groups to investigate this type of heterostructures for future sensor applications with mass production. In addition, there are applications for which cost effectiveness might be less important if efficiency or flexibility is guaranteed. One of the examples is wearable devices.

## 4. Conclusions

In summary, bare Co-rich ribbons without covering and ribbon/Pt heterostructures were considered to explore thermoelectric voltage conversion. In particular, we investigated the Anomalous Nernst Effect and Longitudinal Spin Seebeck Effect in such systems. First, it was observed that the deposition of the very thin Pt layer onto the ribbon surface does not influence the magnetic response of the ferromagnetic amorphous ribbon. Next, we revealed that the thermoelectric results mirror the magnetization curves for a particular magnetic field orientation. In a specific condition, we observed the maximum induced voltage in the thermoelectric experiments. Remarkably, we found that the presence of the Pt layer, beyond protecting against surface oxidation, facilitates the spin-to-charge conversion, increasing the thermoelectric voltage induced in the system. Further, we demonstrated that the employment of thermopiles consisting of Co-rich ribbon/Pt heterostructures in a parallel association increases the thermoelectric current induced in the system, a fact related to the considerable decrease in the electric resistance in the system. Specifically, a linear increase in the thermoelectric current signal was observed with the increase in the number of ribbons in the thermopile, a fact that is relevant for sensor applications, although the number of ribbons can limit the thermopile dimensions. Our findings bring to light noticeable efficiency of the Co-rich ribbon/Pt heterostructure to be used for the conversion of thermoelectric energy through ANE or LSSE.

## Data Availability

Data available from the corresponding author upon reasonable request.

## References

[1] Nykyruy Y., Mudry S., Kulyk Y., Prunitsa V., Borysiuk A. (2023). Magnetic properties and nanocrystallization behavior of Co-based amorphous alloy. Phys. Chem. Solid State.

[2] Murugaiyan P., Mitra A., Panda A.K., Kumar A., Roy R.K., Manna K., Srivastava S.K. (2019). Electromagnetic interference shielding effectiveness of amorphous and nanocomposite soft magnetic ribbons. Phys. B Condens. Matter.

[3] Beach R., Berkowitz A. (1994). Sensitive field-and frequency-dependent impedance spectra of amorphous FeCoSiB wire and ribbon. J. Appl. Phys..

[4] Volchkov S.O., Pasynkova A.A., Derevyanko M.S., Bukreev D.A., Kozlov N.V., Svalov A.V., Semirov A.V. (2021). Magnetoimpedance of CoFeCrSiB Ribbon-Based Sensitive Element with FeNi Covering: Experiment and Modeling. Sensors.

[5] Makhotkin V.E., Shurukhin B.P., Lopatin V.A., Marchukov P.Y., Levin Y.K. (1991). Magnetic field sensors based on amorphous ribbons. Sens. Act. A Phys..

[6] Gazda P., Nowicki M. (2021). Giant Stress-Impedance Effect in CoFeNiMoBSi Alloy in Variation of Applied Magnetic Field. Materials.

[7] Semirov A.V., Bukreev D.A., Moiseev A.A., Derevyanko M.S., Kudryavtsev V.O. (2013). Relationship Between the Temperature Changes of the Magnetostriction Constant and the Impedance of Amorphous Elastically Deformed Soft Magnetic Cobalt-Based Ribbons. Russ. Phys. J..

[8] Kurlyandskaya G.V., Sánchez M.L., Hernando B., Prida V.M., Gorria P., Tejedor M. (2003). Giant-magnetoimpedance-based sensitive element as a model for biosensors. Appl. Phys. Lett..

[9] Yang Z., Lei C., Zhou Y., Cheng Sun X. (2015). Study on the giant magnetoimpedance effect in micro-patterned Co-based amorphous ribbons with single strip structure and tortuous shape. Microsyst. Technol..

[10] Knobel M., Vazquez M., Kraus L., Buschow K.H.J. (2003). Giant magnetoimpedance. Handbook of Magnetic Materials.

[11] Cox C.D.W., Caruana A.J., Cropper M.D., Morrison K. (2020). Anomalous Nernst effect in Co_2_MnSi thin films. J. Phys. D Appl. Phys..

[12] Tian D., Li Y., Qu D., Jin X., Chien C.L. (2015). Separation of spin Seebeck effect and anomalous Nernst effect in Co/Cu/YIG. App. Phys. Lett..

[13] Uchida K., Xiao J., Adachi H., Ohe J., Takahashi S., Ieda J., Ota T., Kajiwara Y., Umezawa H., Kawai H. (2010). Spin Seebeck insulator. Nat. Mater..

[14] Chen T., Minami S., Sakai A., Wang Y., Feng Z., Nomoto T., Hirayama M., Ishii R., Koretsune T., Arita R. (2022). Large anomalous Nernst effect and nodal plane in an iron-based kagome ferromagnet. Sci. Adv..

[15] Melo A.S., de Oliveira A.B., Chesman C., Pace R.D.D., Bohn F., Correa M.A. (2019). Anomalous Nernst effect in stressed magnetostrictive film grown onto flexible substrate. Sci. Rep..

[16] Asaba T., Ivanov V., Thomas S.M., Savrasov S.Y., Thompson J.D., Bauer E.D., Ronning F. (2021). Colossal anomalous Nernst effect in a correlated noncentrosymmetric kagome ferromagnet. Sci. Adv..

[17] Xing Y., Feng Sun Q., Wang J. (2009). Nernst and Seebeck effects in a graphene nanoribbon. Phys. Rev. B.

[18] Correa M.A., Ferreira A., Souza A.L.R., Neto J.M.D., Bohn F., Vaz F., Kurlyandskaya G.V. (2023). Anomalous Nernst Effect in Flexible Co-Based Amorphous Ribbons. Sensors.

[19] Geishendorf K., Vir P., Shekhar C., Felser C., Facio J.I., van den Brink J., Nielsch K., Thomas A., Goennenwein S.T.B. (2020). Signatures of the Magnetic Entropy in the Thermopower Signals in Nanoribbons of the Magnetic Weyl Semimetal Co_3_Sn_2_S_2_. Nano Lett. J..

[20] Kai W., Lin P., Chen W., Kao P., Huang R., Liaw P. (2011). Air-oxidation of a Co-based amorphous ribbon at 400–600 °C. J. Alloys Compd..

[21] Park D., Kim C., Kim W., Hong J. (2007). Study of GMI-valve characteristics in the Co-based amorphous ribbon by ferromagnetic resonance. J. Magn. Magn. Mater..

[22] Egbu J., Ohodnicki P.R., Baltrus J.P., Talaat A., Wright R.F., McHenry M.E. (2022). Analysis of surface roughness and oxidation of FeNi-based metal amorphous nanocomposite alloys. J. Alloys Compd..

[23] Clark A., Zhu A., Sun K., Petty H.R. (2011). Cerium oxide and platinum nanoparticles protect cells from oxidant-mediated apoptosis. J. Nanoparticle Res..

[24] Xu M., Chen F., Wang T., Yu B., Zhao Z., Zhou L., Hua D. (2023). Platinum Nanoparticles Anchored on Covalent Triazine Frameworks Modified Cordierite for Efficient Oxidation of Hydrogen Isotopes. ACS Appl. Nano Mater..

[25] Pragnell W., Evans H., Williams A. (2012). Oxidation protection of Sm_2_Co_17_-based alloys. J. Alloys Compd..

[26] Castel V., Vlietstra N., Youssef J.B., van Wees B.J. (2012). Platinum thickness dependence of the inverse spin-Hall voltage from spin pumping in a hybrid yttrium iron garnet/platinum system. Appl. Phys. Lett..

[27] Sun Y., Chang H., Kabatek M., Song Y.Y., Wang Z., Jantz M., Schneider W., Wu M., Montoya E., Kardasz B. (2013). Damping in Yttrium Iron Garnet Nanoscale Films Capped by Platinum. Phys. Rev. Lett..

[28] Adachi H., Ichi Uchida K., Saitoh E., Maekawa S. (2013). Theory of the spin Seebeck effect. Rep. Prog. Phys..

[29] Snyder G.J., Toberer E.S. (2008). Complex thermoelectric materials. Nat. Mater..

[30] Uchida K., Takahashi S., Harii K., Ieda J., Koshibae W., Ando K., Maekawa S., Saitoh E. (2008). Observation of the spin Seebeck effect. Nature.

[31] Holanda J., Santos O.A., Cunha R.O., Mendes J.B., Rodríguez-Suárez R.L., Azevedo A., Rezende S.M. (2017). Longitudinal spin Seebeck effect in permalloy separated from the anomalous Nernst effect: Theory and experiment. Phys. Rev. B.

[32] Uchida K., Nonaka T., Yoshino T., Kikkawa T., Kikuchi D., Saitoh E. (2012). Enchancement of Spin-Seebeck Voltage by Spin-Hall Thermopile. Appl. Phys. Exp..

[33] Kim J.M., Jeon C.Y., Kim D.J., Van P.C., Jeong J.R., Park B.G. (2020). Amplification of Spin Thermoelectric Signal in Multilayer Spin Thermopiles. ACS Appl. Electron. Mater..

[34] Kim D.J., Lee K.D., Surabhi S., Yoon S.G., Jeong J.R., Park B.G. (2016). Utilization of Antiferromagnetic IrMn electrode in Spin thermoelectric devices and their beneficial hybrid for thermopiles. Adv. Func. Mater..

[35] Weng T.W., Chuang T.C., Qu D., Huang S.Y. (2022). Anomalous Nernst thermopile made of single element iron. J. Magn. Magn. Mater..

[36] Fal Miyar V., Cerdeira M.A., García J.A., Potatov A.P., Pierna A.R., Marzo F.F., Barandiarán J.M., Kurlyandskaya G.V. (2008). Giant magnetoimpedance of electrochemically surface modified Co-based amorphous ribbons. IEEE Trans. Magn..

[37] Kurlyandskaya G.V., Lezama L., Pasynkova A.A., Volchkov S.O., Lukshina V.A., Larrañaga A., Dmitrieva N.V., Timofeeva A.V., Orue I. (2022). Amorphous FeCoCrSiB Ribbons with Tailored Anisotropy for the Development of Magnetic Elements for High Frequency Applications. Materials.

[38] Kurlyandskaya G., Dmitrieva N., Zayarnaya T., Lukshina V., Potapov A. (1996). The thermomechanical treatment of an amorphous Co-based alloy with a low Curie temperature. J. Magn. Magn. Mater..

[39] Althammer M., Meyer S., Nakayama H., Schreier M., Altmannshofer S., Weiler M., Huebl H., Geprãgs S., Opel M., Gross R. (2013). Quantitative study of the spin Hall magnetoresistance in ferromagnetic insulator/normal metal hybrids. Phys. Rev. B.

[40] Melzer M., Kaltenbrunner M., Makarov D., Karnaushenko D., Karnaushenko D., Sekitani T., Someya T., Schmidt O.G. (2015). Imperceptible magnetoelectronics. Nat. Commun..

